# “It was like walking without knowing where I was going”: A Qualitative Study of Autism in a UK Somali Migrant Community

**DOI:** 10.1007/s10803-016-2952-9

**Published:** 2016-11-17

**Authors:** Fiona Fox, Nura Aabe, Katrina Turner, Sabi Redwood, Dheeraj Rai

**Affiliations:** 1 The National Institute for Health Research Collaboration for Leadership in Applied Health Research and Care West (NIHR CLAHRC West), University Hospitals Bristol NHS Foundation Trust, 9th floor, Whitefriars, Lewins Mead, Bristol, BS1 2NT UK; 2 School of Social and Community Medicine, University of Bristol, Canynge Hall, 39 Whatley Road, Bristol, BS8 2PS UK; 3 Autism Independence, Silai Centre, 176 Easton Road, Bristol, BS5 0ES UK; 4 Bristol Autism Spectrum Service, Avon & Wiltshire Partnership NHS Mental Health Trust, 3 Petherton Road, Bristol, BS14 9BP UK

**Keywords:** Autism, Somali, Migrant, Qualitative, Attitudes, Childhood, Disability, Help-seeking

## Abstract

Increasing recognition of autism in Somali migrant communities means that appropriate support services are needed. Attitudes to autism and barriers related to help-seeking in these communities are poorly understood. We aimed to assess what families affected by autism need, and how health, education and social care services can support them. In partnership with the local Somali community the research team conducted 15 in-depth interviews with parents affected by autism. Two themes are reported; ‘Perceptions of Autism’ and ‘Navigating the System’. Our research shows the importance of understanding cultural views of autism and the need to raise awareness, reduce stigma and provide support to encourage families not to delay seeking help for their children.

## Introduction

Several large population based studies have suggested that autism may be over-represented in some migrant groups in Western countries, such as the Somalis (Magnusson et al. 2012; Hewitt et al. 2013; Bolton et al. 2014; van der Ven et al. 2013; Becerra et al. 2014; Barnevik-Olsson et al. 2010; Lehti et al. 2013). The reasons for a higher prevalence of autism in these groups are still unclear, but it is acknowledged that immigrant populations require appropriate help and support in relation to autism services (Hewitt et al. 2013). Despite this need, it is also known that migrant populations are less likely to access health services, particularly those providing support for mental health or developmental disorders (Edbrooke-Childs et al. 2016; Teunissen et al. 2014; Fassaert et al. 2009). However, early diagnosis and intervention may lead to improved developmental trajectory and outcomes (Fernell et al. 2013).

The Somali migrant population in the UK largely comprises first generation migrants who sought refuge following the civil war in Somalia since the 1990s, and their descendants who were born in Britain. Nationally, Somali communities have one of the highest rates amongst migrant groups of poverty, unemployment and social and educational inequalities (Jabłonowski et al. 2013). The Somali population are the second largest migrant community in Bristol and the fourth largest Somali community among British local authorities (Bristol City Council 2013). Precarious employment and high health needs are characteristic of the Bristol Somali community, and are exacerbated by low English skills, limited access to training and lack of transferable skills (Jabłonowski et al. 2013). To our knowledge, there have been no published reports on the prevalence, the clinical diagnosis or community attitudes towards autism in Somalia. Furthermore, not much is known about the needs of the Somali migrant population raising children with autism, their experiences of the diagnostic process, barriers to care, culture specific aspects of seeking help from health, or education and social care services. Such information is essential in order to provide high quality services and reduce inequalities in care for this group.

The aim of this qualitative study was to address this gap in knowledge, in partnership with members of the Somali community in Bristol, to gain a better understanding of their perceptions, experiences, and support needs. Through in-depth interviews we explored how autism is seen and understood, how parents find out that their child has autism, and their experiences of accessing health, education and social care services.

## Methods

### Study Design

We adopted a community-based participatory research (CBPR) approach which is underpinned by principles of community engagement and empowerment, mutual respect and co-learning, as well as commitments to action and improvement (Minkler and Wallerstein 2010; Wright et al. 2014). This approach to research represents an attempt to reduce health inequalities in groups who are often marginalised with few material and social resources to draw on.

Our study was conducted in partnership with a community organisation, Autism Independence (AI), which is a network of Somali parents who have children with autism. Their aim is to raise awareness of autism, and to educate and empower affected people and their families, especially those with no or little previous knowledge about autism and those with limited access to health and social care services. Members of AI were involved in all aspects of the research, from specifying research questions to helping conduct the research, developing recommendations on the basis of the findings, and dissemination. The approach, study design and methods were negotiated between the study team and members of AI. Author NA, who founded AI and was a member of the study team, provided ‘cultural brokerage’ between the study team and the local Somali community, and during initial meetings mediated between potential participants’ enthusiasm for quick action and improvement, and the slower pace required for research processes. Research materials, including information flyers, and the interview schedule were developed and produced both in English and Somali. A favourable ethical opinion was obtained from the Faculty of Medicine and Dentistry Research Ethics Committee, University of Bristol.

The mutually agreed aims of the research were to develop a clearer and more nuanced understanding of the range of views on and perceptions of (1) autism in the local Somali community, (2) the process through which a child was identified as having autism, and (3) the experiences and challenges of accessing and engaging with services, including suggestions about how the process of diagnosis and receiving services could be improved to fit more closely with social and cultural needs. Due to the study context and its exploratory and collaborative nature, a qualitative, interview-based approach was selected.

### Recruitment and Participants

Purposive sampling was used to achieve a maximum variation sample (Patton 2005; Phillimore et al. 2014). Inclusion criteria were: (1) being a parent to a child under 16 years of age, who has a diagnosis of autism and (2) identifying as a member of the Bristol Somali migrant community. If these two criteria were met, there were no exclusion criteria.

An invitation to participate was first sent to a social media group for AI attendees, coordinated by NA. Nine parents expressed an interest and subsequently took part in an interview. After this initial phase of interviewing, the study steering group discussed the sample characteristics. In order to achieve maximum variation, the study steering group advised targeted sampling of parents of older children and male participants (fathers), since these specific groups were under-represented in the sample. In a further round of recruitment, Somali families known to AI, who had a child with autism were contacted directly by NA and invited to participate. Six further parents agreed to take part, leading to a total of 15 in depth interviews. This number was deemed appropriate for the exploratory qualitative design and to meet the study aims. All participants were given a participant information sheet in both Somali and English in advance, and full understanding was checked before interviews began. Written consent was sought to audio-record and fully transcribe the interviews.

Of the parent participants, 12 were female and three were male and all were from separate families. Their average age was 36 years. Between them the participants had 17 children with a diagnosis of autism, of whom five were girls and 12 were boys. Two families each had two children with autism (twins aged 4 years, and sisters aged 9 and 5 years respectively). The children’s ages ranged from 4 to 13 years (average 7 years). Six of the 17 children were described as completely non-verbal (four of whom were under the age of 5 years) and the others ranged from speaking a little, to having full speech. The characteristics of the sample are provided in Table 1.

**Table 1:** Sample Characteristics

Participant ID	Gender	Age group	Relation-ship status	Arrival in UK	Age of child with autism	Total no of children: position of child with autism	Severity	Language interview conducted in
PPT1	F	26–30	Separated	2001	4	5, 3rd	Non-verbal	Somali
PPT2	M	36–40	Married	2006	6	4, 2nd	Non-verbal	English
PPT3	F	46–50	Married	2007	12	7, 5th	Started talking	Somali
PPT4	M	56–60	Married	1999	13	6, 4th	Non-verbal	English
PPT5	F	36–40	Married	2005	7	7, 5th	Started talking	Somali
PPT6	F	26–30	Married	2007	5	3, 1st	Non-verbal	Both
PPT7	F	31–35	Separated	2004	7	3, 2nd	Started talking	English
PPT8	M	26–30	Separated	2012	5	3, 1st	Non-verbal	English
PPT9	F	31–35	Married	2005	6	4, 3rd	Talks a little	Both
PPT10	F	46–50	Married	1992	14	8, 5th	Talks	Somali
PPT11	F	26–30	Married	2009	4, 4	4, 1st (twins)	One talks more	Somali
PPT12	F	31–35	Married	2004	8	4, 2nd	Talks	Somali
PPT13	F	41–45	Married	2002	9, 5	5, 3rd and 5th	Older talks more	Somali
PPT14	F	36–40	Separated	2003	9	7, 3rd	Non-verbal	Somali
PPT15	F	26–30	Married	2008	5	3, 2nd	Talks a little	English

### Data Collection

Interviews took place between July and September 2015, at a community centre or in participants’ own homes, according to their preference. Interviews were guided by a semi-structured interview schedule, developed by the research team and refined following a pilot interview with a Somali mother, whose data were not included in the analysis. FF led the questions and NA provided translation for those who wished to speak in Somali. NA also used prompts to elicit greater detail and concrete descriptions of experiences. Interviews lasted 45–95 min and explored family’s experiences of having a child with autism, from the first time they became aware of their child’s difference, through the process of diagnosis and their subsequent experiences of health, social and education services. The interviews were audio recorded and a professional company transcribed the spoken English. NA then audio checked the transcripts for accuracy, adding any passages where the spoken Somali was not fully translated during the interview. FF anonymised the transcripts prior to analysis.

### Analysis

Interview data, fieldwork notes and debriefing notes following each interview were analysed using inductive thematic analysis (Braun and Clarke 2006), involving initial coding, the forming and refining of categories, searching for negative evidence and comparison across the data set at each stage of the analysis. After the first three interviews, FF and NA identified codes in the transcripts and discussed, refined and agreed on these codes. From this a thematic coding framework was drafted, which was then discussed with the wider study team, who also had access to the raw data. NVivo software (QSR International Pty Ltd 2012) was then used for data management, and new codes were added to the coding framework as they arose. During this process, the coding structure was revised, merged and refined to develop a coherent thematic summary which was discussed and agreed by the study team.

## Results

**Fig. 1:**
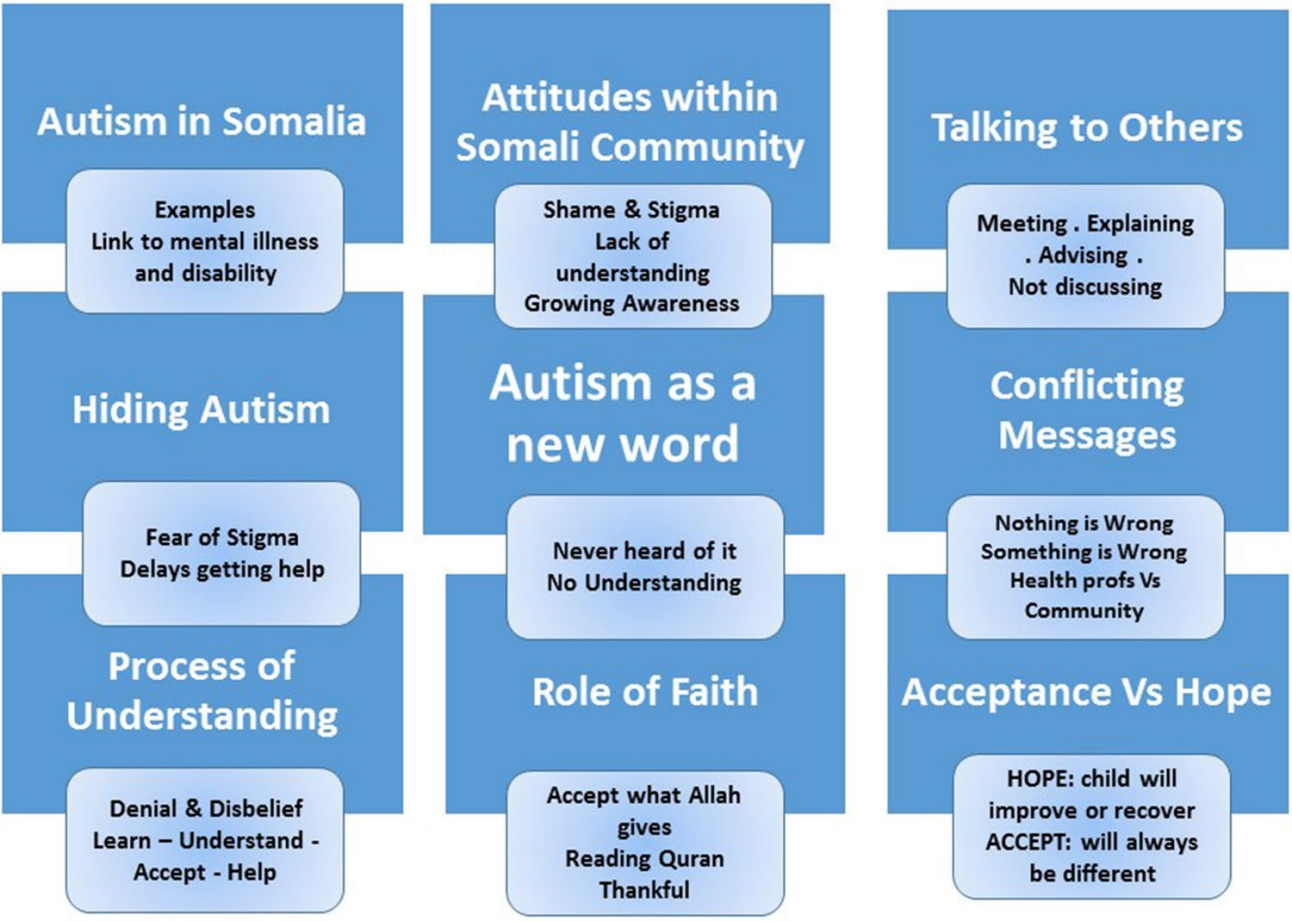
Perceptions of Autism

Four major themes were identified through analysis; ‘My child is different’; ‘Perceptions of Autism’; ‘Navigating the System’ and ‘Support’. Due to limited space two themes; ‘Perceptions of Autism’ and ‘Navigating the System’ are presented here. These have been selected because they specifically illuminate the social and cultural context which affect acceptance of autism and help-seeking. Interview excerpts are presented to provide nuanced illustrations of each theme (Fig. 1).

### A New Word

Many participants explained that when they were told their child had autism, this was an unfamiliar word to them, and they were left feeling shocked and confused. They highlighted the lack of a Somali word for autism and many indicated that autism is not recognised in Somalia:

“People in Somalia have not heard of autism before. No, they haven’t. There is no autistic person in Somalia. There isn’t anybody who doesn’t talk in Somalia. I haven’t seen anybody. And people said they haven’t seen anyone who don’t talk and have something like autism in Somalia” (PPT 12).

The lack of physical impairment was also a source of confusion for participants, who frequently cited their difficulty reconciling the mismatch between a child who looks ‘normal’ yet has difficulties with communication, social interaction and behaviour. In the participants’ experience, a serious disorder would be accompanied by physical signs of disability:

“I have not come across a child that looks so normal as my son, but yet is different and it’s the way he is. I come across children who are physically disabled, who look different in their face, and you can see they are disabled, but I’ve never come across a disabled child like X*” (PPT 6).

This lack of vocabulary, combined with uncertainty around the status of autism as a mental illness, or disability, made it difficult for participants to communicate with their own extended families and community, who often did not understand or accept the child’s diagnosis.

### Hiding their Child with Autism

Challenging behaviours that were common among the children in our sample, such as running away and violent outbursts, were not tolerated by their local community;

“Sometimes, when you’re walking or even in the playground or somewhere, he’ll hit any child”. Like, even if you say sorry… Last week, he hit a man who was sitting, and I told him, “Sorry” twice. He said, “No, no, he hit me.” I said, “Sorry.” There was another lady who already saw when he was hitting, she told me, “There’s a man who’s going to call the police for your son.” (PPT 15). Consequently, some families avoided public places with their child. In addition, negative perceptions of mental illness within the Somali community meant that many families experienced stigma and social exclusion:

“There are a few people who think that I have got a child who is mentally ill, and that I shouldn’t go near them. I don’t like to go to certain people’s houses because I don’t feel they sympathise, or I don’t feel confident enough to go to their houses” (PPT 7).

This stigma reinforced families’ tendency to hide their child and to delay help-seeking. The extent to which participants discussed autism with other people varied according to their stage of acceptance and their concerns about stigma. Some participants avoided interacting with neighbours or friends, and many reported that other Somali families hide their children with autism and were unwilling to talk about them. This reluctance was seen as detrimental, as it prevented getting help early for their children. On the other hand, many families in our sample talked about their children with great pride and expressed that they were not ashamed of their child’s autism:

“I’m not the kind of person who hides my child and I’m not afraid; I’m not ashamed that my son has autism... I am not, but there are people out there who are ashamed. People hide; people say, ‘Don’t tell other people that my son has autism’… I don’t care what other people think. I’ve got my normal children and my son has autism. I don’t care what people think.” (PPT 14).

### Conflicting Messages

When participants did share their child’s diagnosis, it was common for members of their community and extended family (both in Somalia and resident in the UK) to tell them not to worry, to disregard the diagnosis and to persuade them that their child would be fine:

“Some of the people say, “Why are you saying something silly like this?” He’s a child, he will grow out of it. A lot of children can’t talk at the normal age, why don’t you wait? Don’t go to the doctors. He will grow out of these things. Because he’s so young, he will come up later on.” (PPT 9).

“My family and my community said, “There’s nothing wrong with him,” and, “Later on, at some stage, when he grows up, he will talk. He’s just got delayed language, and everything will be fine.” People were pushing me to believe that there was nothing wrong with him” (PPT 7).

Advice given by members of their families and community, to help the child to recover or improve, included taking the child to Somalia, or giving them camel milk;

“You often get told too many ideas when people see you in the community, that you’ve got a child with autism”… there are the ones who say, “Take your child back home. That would help them, or give them camel milk. That will help them” (PPT 1).

Participants’ accounts indicated how conflicting messages from healthcare professionals and their community contributed to their confusion about the difference between autism as a long term condition, or temporary developmental delay:

“So there are the people who tell me in the Somali community, “He will get better and that he would make progress and that he would grow out of autism”. And there are the other ones who say “It will be with him for the rest of his life”. There was a Somali doctor on the Somali TV who said there are some who grow out of it. So I don’t understand, I am confused” (PPT 5).

### Acceptance versus Hope

Community attitudes towards mental illness, challenging behaviours and disability, combined with the lack of vocabulary to describe and explain autism, made acceptance extremely challenging. It was therefore difficult for parents to recognise that their child’s disability was characteristic of autism. These attitudes also prevented parents from sharing their concerns about their child, meaning that assessment and diagnosis were sometimes delayed:

“I kept it to myself a lot. I thought, if I had the right people at the right time, and I took him to a nursery at an early age, because he was entitled to it. He is a special child. But I wasn’t convinced to tell anybody or to share with anyone what’s going on” (PPT 7).

In our sample the extent to which parents accepted their child’s diagnosis varied between and within families, and discrepancies between some couples were evident:

“He (father) doesn’t believe there’s anything wrong them ... He believes that they have autism, but little autism. He doesn’t believe that there is something wrong, there’s something hugely wrong with them. And I worry a lot. And I don’t trust. There is obviously something wrong with them.” (PPT 11, mother of twins).

One mother reported how she requested an assessment for the child against her husband’s wishes;

“We’ve been apart for that period because we had different ideas about him. He always convinced me, “He will grow out of it. Don’t go to the doctors.” He has that opinion. I am the person who called the health visitors in. He wasn’t even aware. I didn’t tell him that I’m going to. When she knocked on our door, he realised that we will have a health visitor visiting us on that morning. When he realised that something was going on, it was when we were on the assessment.” (PPT 9).

Although four participants were separated from their spouse, they were reluctant to discuss the reasons for their separation. However, one participant spoke about the connection between her son’s autism and the breakdown of her marriage:

“He left because of my son’s autism. He didn’t want to look after, he didn’t want to help; he didn’t want to look after him, not even one night.” He… started eating khat, this leaf thing that Somali people eat, like a drug. He left because he didn’t want to be part of it; he didn’t want to be… His son was up every night, and I was up every night and he said, “I’m not interested.” So, I said then, “If you’re not going to help me with him, then you can leave.” (PPT 14).

Faith played a crucial role in acceptance, and participants described Allah as being in control of their lives. They frequently expressed gratitude and trust in Allah’s plan for the future and indicated that their faith was a source of comfort, helping them to cope:

“Well, it’s like when you are Muslim, you have to believe whatever happens to you that it’s not coming from you. It’s not your fault. It’s what Allah already wrote and then you have to accept. So it’s the child’s difference.” (PPT 2).

Conversely, faith fostered hope that their child will improve, or ‘get better’ in the future, creating a tension between acceptance and hope that, with Allah’s help things might also change and that their child might improve or recover:

“I am hoping Allah will help, one day he will get better. I do a lot of praying that Allah will help him. With Allah, some of our prayers that we have been doing will help us, when we can see that he has been making progress. I pray to Allah that things will change for *X, for the better. We believe that Allah God can change anything.” (PPT 5).

Observing progress in their child’s development also led some parents to feel hopeful that their child did not have Autism, or might grow out of it;

*“*They say that now that he’s talking and he’s asking what he wants, he probably will be fine. Maybe when he grows up, he will be fine” (PPT 12).

### Learning and Understanding

The process of accepting their child’s diagnosis required understanding of autism which, in turn, required learning more about it. The explanation of autism offered at the point of diagnosis was often deemed to be inadequate and difficult to understand:

“So, to understand what it meant would have made so much difference – a way that I could understand what was wrong with *X. I wish I had more information and better explanation of what autism meant. It was like walking without knowing where I was going.” (PPT 12).

Some participants expressed uncertainty about the diagnosis that included unfamiliar cultural milestones, such as making eye contact:

*“*The doctor, he said, “He doesn’t have eye contact.” I said, “What does that mean, eye contact?” Then, really I noticed he’s different, but I don’t understand the autism, the meaning. I’d never seen children the same as my son, but really he’s different.” (PPT 7).

The extent to which parents were given adequate information about autism varied greatly, from no information at all, to written information provided in Somali. The provision of information was often overlooked in favour of focusing instead on how to help the child and organising follow up appointments. Participants therefore developed their own strategies to gather information and learn about autism. These included accessing information online, learning through professionals who they encountered after diagnosis, and attending a parent support programme. The latter was especially supportive in terms of learning through interaction with other families:

“I met other Somali families that I can speak my own language with who have children same as mine. They’ve experienced how their kids are. I was speaking to them in my own language, I felt comfortable when I came back and relieved by talking to all these families. There was an interpreter and it was explained, the learning. Some of the things we were learning could help you how to deal when they misbehave with their parents. I’ve learnt quite a number of things that we didn’t know before.” (PPT 13).

Some participants drew on a combination of these sources to increase their understanding and knowledge of autism:

“I had some leaflets, some information about autism. I was given a website that was in Somali that explained about autism. I go to some other groups that talk about autism. I went to different talks about autism and sometimes they talk about the support from social care, from different things, and I have spoken to different doctors.” (PPT 11).

**Fig. 2:**
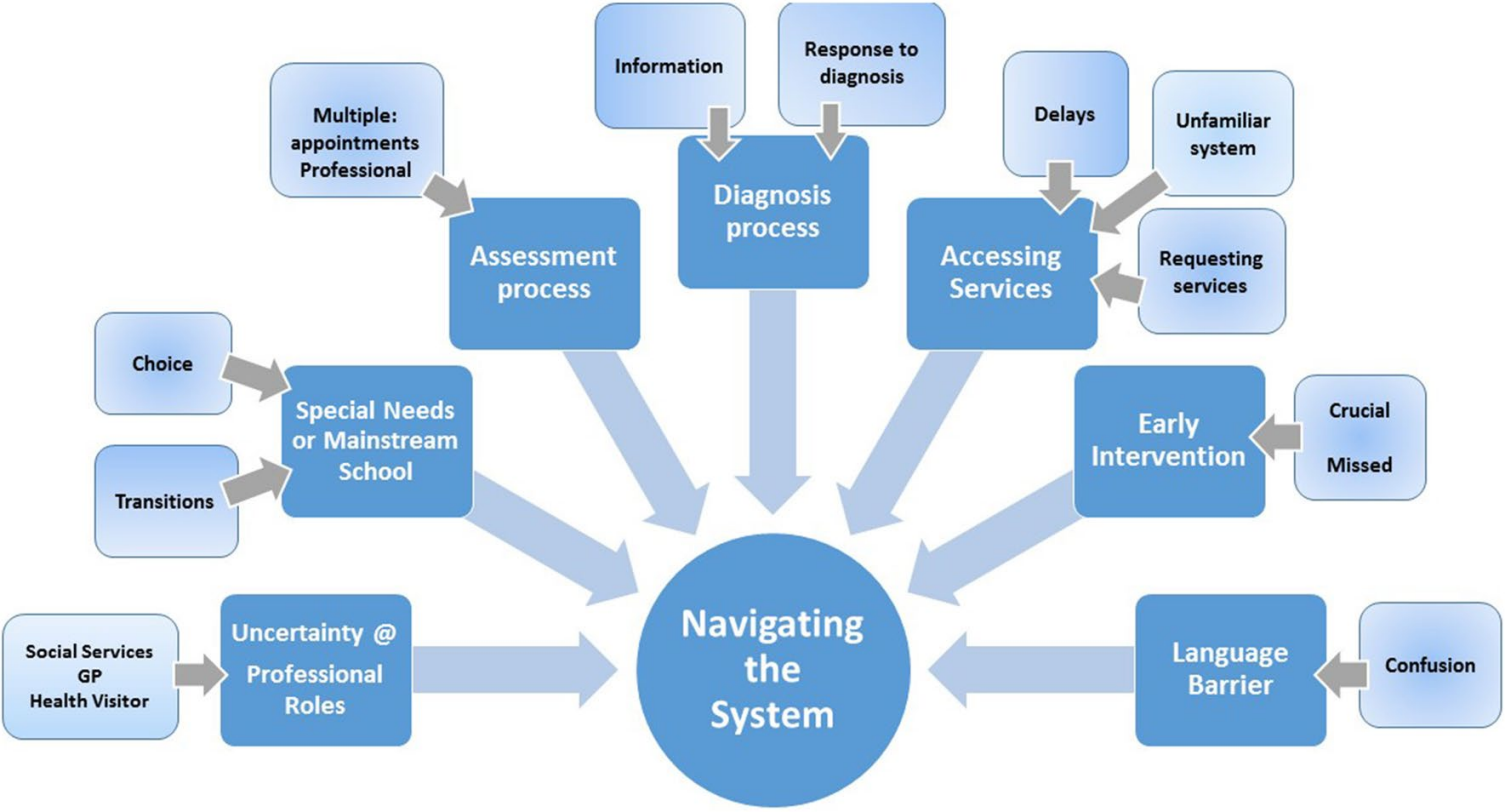
Navigating the System

Once parents began to learn about autism, they were able to use the unfamiliar vocabulary when speaking to others and increase their own understanding;

“It’s only recently I’m beginning to understand and talk about the autism; I knew there was something wrong because of her crying, but not really talked about it.” (PPT 1) (Fig. 2).

### Assessment and Diagnosis

Parents in our sample reported having noticed that their child was ‘different’ to, or not developing in the same way as other children, but only some proactively sought advice from their health visitor;

“I told my health visitor.” I phoned my health visitor and told them, “My son is not sitting properly. He doesn’t talk. He’s different from my other kids. What’s going on?” (PPT 10).

Others were alerted to their child’s developmental delay by nursery staff. Either way, the process of assessment for their child was usually mediated by health visitors and nursery schools who made the appropriate referrals. This was described as a period of uncertainty, where the purpose of appointments and professional roles were not always clear to the parent;

“There were different people who used to observe X* in nursery. They had notebooks and were writing down information as X* came to the nursery. I didn’t know exactly who they were, but I’m assuming it was this lady who has sent them to observe them to maybe make the decision of what was wrong with her” (PPT 1).

The diagnosis of autism was typically confirmed in multi-disciplinary meetings, and feelings of shock, confusion, denial, upset and sadness were common;

*‘*That day I asked the doctor, “Will autism be with her for the rest of her life? Is it something that she’s going to grow out of?” She said, “No,” it was going to be with her for the rest of her life. I was very shocked and the doctor could see how upset, how sad I was about her diagnosis’ (PPT 1).

### Accessing Services

Accessing health and education services may be challenging for any family in the period after their child’s diagnosis. Our findings indicated that this may be intensified for those to whom the system is unfamiliar and for whom English is not the first language:

“I never had the opportunity to learn and understand the system or learn the language, so there is a barrier of not understanding how this whole system works and operates around schools, around different services.” (PPT 1).

Even participants who were proficient English speakers reported that terms or words used were incomprehensible to them. A father who spoke fluent English commented:

“You have to do it by yourself. But if you are not doing good what you want to do and you are not thinking and sometimes you can get more terminology words on their reports or sometimes you cannot understand it, you have to get a dictionary, whatever it is.” (PPT 2).

Several participants identified times where an interpreter, or a specialist Somali support worker, would have enhanced their understanding of the process of assessment, diagnosis and engaging with services:

“I would have loved to have seen some people from a Somali background who are doing this, who are making family members understand a little bit more about autism, especially the ones that have a language barrier, which I think, 70 to 60 % of them do have that problem.” (PPT 8).

Some reported feeling overwhelmed by their lack of understanding about the health and education system and many did not know how or where to start to access help. Uncertainty about professional roles and confusion about the number and purpose of appointments filtered through the participant’s accounts:

“I think she’s a social worker. She’s like a… What was her title? She was… Our doctor tell us she’s a co-worker and she works with children with autism.” (PPT2).

The lack of familiarity, in combination with a fragmented system and the language barrier all contributed to delays for some families in accessing support for their child. This was a source of frustration for some participants, who expressed that earlier intervention would have been helpful:

“When you’re new to a country, it’s just impossible for you to find all the different services available. The reason that causes further delay to our children or [accessing] services is because you haven’t got the capacity, or the ability, or the understanding to access services … That’s what causes, I think, a lot of the delays.” (PPT 3).

“Yes, the earlier it is done, the better, because I can see, if my child would have had a teacher when he was two years of age, or assistance towards language skills, towards communication skills. He is five, so three years could have made a difference, because we had to wait all the way until he was four years old, school age.” (PPT 8).

Many participants felt that they needed more support to understand and access the services available. It was identified that a Somali link worker could help to bridge the language barrier and address cultural understanding of autism:

“If there could be any healthcare assistants, or maybe any social workers or anyone with a Somali background who knows about autism, or if they don’t know, anyone to be trained for that, so they can provide the help that the families need, that would have been something big. That would be something that makes a difference, to be frankly honest with you, to the children’s lives, the families’ lives.” (PPT 8).

### Social Services

Attitudes towards the support offered by social services varied. Some participants reported that they realised what an important source of help social services could be, but were frustrated with delays in receiving support, such as accessing benefits, or home modifications:

“I’ve tried to contact social services, but I haven’t had one yet. It’s been a very long wait ... Yes. I can easily say, without the social services you can’t have anything.” (PPT 9).

Some accounts however, revealed a mistrust of social services and this affected the participants’ willingness to engage with social care. There was widespread concern that their child would be taken away from them if they were seen to be having difficulty coping with the demands of raising a child with autism. One participant told the story of a woman whose child was removed, which had fuelled much alarm:

“Yes, X’s story impacted all families in the Somali community who have a child with autism. There’s the fear that their child will get taken away from them because of what happened to X. Although they help you, that fear at the back of your mind is that they’re watching you and that they will take away from you.” (PPT 14).

### Education Services

Securing the most appropriate educational provision was particularly challenging and was fraught with issues around lack of choice and transitions between nursery and school. Many parents had strong preferences about mainstream or special needs schools, depending upon the specific needs of their child. Reasons for the former included not wanting their child to be with more severely affected children, preferring them to learn alongside peers who could act as role models. Some parents wanted their child to attend a special needs school that catered specifically for children with communication disorders. Sometimes this seemed to reflect a wish to distance their child from other children with physical or mental disabilities;

“I would like to look around a special needs [school] and see. I don’t want special needs schools that cater for all disabilities, like children with wheelchairs. I would like somewhere that helps her with communication, so being part of a group of children who have got, for example, language difficulties only.” (PPT 1).

Some participants were appreciative of the advice about the best school for their child that they received from staff at school and nursery, or Special Educational Needs Coordinators (SENCO). Nursey staff were often identified as a key source of support, referring families to sleep programs, or reminding parents to keep appointments and trusting relationships developed. Good communication was a key factor in effective partnerships between parents and educational staff. This was often mediated through a diary of the child’s activities that was passed from school to home, or via regular meetings with the child’s one to one workers;

“Yes, so I take someone to help me with the language, an interpreter, and we do work together (with the key workers). I ask of them questions and we work together.” (PPT 3).

## Discussion

To our knowledge, this is the first in depth study of the perceptions of autism and challenges to help-seeking in a Somali population within the UK. The qualitative data generated through interviews and the inductive analysis facilitated a nuanced insight into local experiences that appear consistent with the findings of the few studies of Somali communities conducted elsewhere (Minnesota 2014; Miller-Gairy and Mofya 2015). For the Somali families in our study, cultural attitudes towards mental illness, challenging behaviour and disability, combined with the lack of vocabulary to describe and explain autism made the understanding and acceptance of their child’s autism particularly difficult. Evidence suggests that mental illness is stigmatized in Somali culture and the existence of some conditions may even be denied altogether (Elmi 1999). Our findings support evidence that the perceived link to mental illness or ‘insanity’ mean that families affected by autism experience stigma and social isolation (Selman et al. 2016; Schuchman and McDonald 2004; Minnesota 2014). Parents in our study also reported that stigma related to mental health, challenging behaviour and disability reinforced families’ tendency to hide their child and to avoid seeking help early.

Parents often demonstrated uncertainty about the long-term nature of autism and this was affected by three key factors. First, lay advice from their families and community encouraged parents to believe that their child would ‘improve’ and reassured them that they should not worry, or seek professional help. Advice, such as taking the child home to Somalia, or not worrying, often conflicted with the advice of professionals, leaving parents feeling confused and isolated in their decision-making. Second, witnessing the developmental progress made by their children corroborated lay advice and gave some parents hope that their child might ‘grow out’ of autism, or might not have autism at all. Third, and linked to this, was a tension between acceptance and hope, fostered by their faith that their child could improve, or ‘get better’ in the future, which was a source of comfort. Our work suggests that the word ‘autism’, the range of neurodevelopmental symptoms that it denotes, often in the absence of physical disability, and its long term trajectory require careful and culturally sensitive explanations. Strategies for increasing awareness and understanding about autism within the Somali community could be designed and informed by community members (Minnesota 2014) and delivered via a range of community channels to effectively reach all family members and the wider community. Community-led organisations may play a crucial role in engaging and supporting parents whose children have autism.

Our findings illustrate the support needed by many Somali families from the pre-diagnosis period of assessment, to understand the purposes of appointments and the roles of professionals. Where trusting relationships developed with healthcare professionals or school staff, these needs were often met. However, without such support children missed out on crucial early intervention and parents lost the support they need to cope with conflicting messages, stigma and social isolation. The lack of knowledge about autism within their community meant that parents had to negotiate their own process of understanding and acceptance within the context of conflicting advice from their friends and family.

There is some previous evidence to suggest that Somali parents commonly react to an autism diagnosis with denial (Kuenzli 2012). We found that parents accepted their child’s diagnosis to varying degrees and within families this may be a potential source of tension. Our analysis identified some examples where fathers were disengaged and unsupportive, or had left the family altogether. By contrast, the three fathers who took part in interviews were engaged in learning about autism, supporting their child and sharing their knowledge informally with others. This highlighted a potential opportunity to work with men who are positive about fathering children with autism, to develop their role as peer supporters for other Somali men affected by autism.

Some parents in our study undertook their own enquiries to better understand their child’s diagnosis and to access support. However, our findings indicate how bewildering it can be for migrant populations to understand and navigate unfamiliar health, education and social care systems, even for those who spoke fluent English. This experience was echoed by US research where the major barriers to accessing services were reported to be a lack of knowledge about and fragmentation of services (Kuenzli 2012). The language barrier was identified as especially pertinent to this problem (Minnesota 2014). Beyond the language barrier, our findings also suggest that community perceptions of autism can challenge parents’ ability to understand and accept their child’s diagnosis. While participants in the study described their efforts to ensure appropriate support for their child, many experienced frustrating delays.

Our findings indicate that support is required to help families to negotiate and navigate the health, social and educational services available to them during assessment, diagnosis and beyond. Some participants advocated that this role could be fulfilled by a link worker, ideally from the community or who speaks Somali and who has comprehensive understanding of autism, to support and guide families through the confusing early stages of understanding and acceptance.

### Strengths and Limitations

The community-based participatory approach ensured that the design and conduct of the research was, at all stages, culturally sensitive and responsive to the needs of the local Somali community. The co-production of this study ensured that members of this often hard-to-reach group were actively engaged in the research. In-depth interviews were facilitated by co-interviewers in both English and Somali, which led to detailed and often moving personal accounts, and some participants expressed that they valued the opportunity to tell their story. Experienced qualitative researchers (FF, SR and KT) worked closely with co-researcher NA to ensure that the inductive analysis reflected the participants’ views and experiences.

This was a small scale, exploratory qualitative study and as such, the findings should be considered as an insight into the views and experiences of a Somali Community in one UK city, although they do mirror evidence from international research (Minnesota 2014; Miller-Gairy and Mofya 2015). It should be noted that the first nine parents all volunteered to participate and were therefore self-selecting and were engaged with AI and thus had more access to information and support from other parents raising a child with autism. We made more proactive efforts to ensure participation of the other six parents, who were less engaged with the AI network. Despite best efforts, we struggled to recruit many fathers into the study, which may reflect a wider reluctance of Somali men to discuss their child’s autism. Some participants seemed reluctant to discuss the extent of their difficulties, even though the interview was carried with a member of their own community. This may reflect mistrust about the purpose of the research. With her dual roles as researcher and community worker, NA was sometimes aware of such discrepancies but always maintained confidentiality. Our sample included families with children aged 4–14 years of age. Whilst this enabled us to focus on the experiences of assessment and diagnosis, it did preclude exploration of issues pertinent to older children. Future research could focus on Somali families with older adolescent or adult children in order to gain a fuller understanding of their ongoing needs.

## Conclusion

Our findings highlight the importance of culture specific issues in autism and the challenges and barriers that Somali parents encounter in understanding and accepting autism and accessing appropriate support. These findings suggest the need to increase understanding via range of community channels in order to raise awareness, reduce stigma and provide support to encourage families not to delay seeking help for their children.
